# VEROnA Protocol: A Pilot, Open-Label, Single-Arm, Phase 0, Window-of-Opportunity Study of Vandetanib-Eluting Radiopaque Embolic Beads (BTG-002814) in Patients With Resectable Liver Malignancies

**DOI:** 10.2196/13696

**Published:** 2019-10-02

**Authors:** Laura Beaton, Henry F J Tregidgo, Sami A Znati, Sharon Forsyth, Matthew J Clarkson, Steven Bandula, Manil Chouhan, Helen L Lowe, May Zaw Thin, Julian Hague, Dinesh Sharma, Joerg-Matthias Pollok, Brian R Davidson, Jowad Raja, Graham Munneke, Daniel J Stuckey, Zainab A Bascal, Paul E Wilde, Sarah Cooper, Samantha Ryan, Peter Czuczman, Eveline Boucher, John A Hartley, Andrew L Lewis, Marnix Jansen, Tim Meyer, Ricky A Sharma

**Affiliations:** 1 University College London Cancer Institute, University College London London United Kingdom; 2 Department of Medical Physics and Biomedical Engineering, University College London London United Kingdom; 3 Cancer Research UK University College London Cancer Trials Centre London United Kingdom; 4 University College London Centre for Medical Imaging, University College London London United Kingdom; 5 University College London Experimental Cancer Medicine Centre Good Clinical Laboratory Practice Facility University College London London United Kingdom; 6 Centre for Advanced Biomedical Imaging University College London London United Kingdom; 7 University College London Hospitals NHS Foundation Trust London United Kingdom; 8 Division of Transplantation and Immunology, Royal Free Hospital NHS Foundation Trust London United Kingdom; 9 Division of Surgery and Interventional Science, University College London London United Kingdom; 10 Hepatopancreatobiliary Surgery and Liver Transplantation, Royal Free Hospital NHS Foundation Trust London United Kingdom; 11 Biocompatibles UK Ltd London United Kingdom; 12 Department of Oncology, Royal Free Hospital NHS Foundation Trust London United Kingdom; 13 National Institute for Health Research University College London Hospitals Biomedical Centre University College London Cancer Institute London United Kingdom

**Keywords:** hepatocellular carcinoma, metastatic colorectal cancer, liver metastases, transarterial chemoembolization vandetanib

## Abstract

**Background:**

Transarterial chemoembolization (TACE) is the current standard of care for patients with intermediate-stage hepatocellular carcinoma (HCC) and is also a treatment option for patients with liver metastases from colorectal cancer. However, TACE is not a curative treatment, and tumor progression occurs in more than half of the patients treated. Despite advances and technical refinements of TACE, including the introduction of drug-eluting beads-TACE, the clinical efficacy of TACE has not been optimized, and improved arterial therapies are required.

**Objective:**

The primary objectives of the VEROnA study are to evaluate the safety and tolerability of vandetanib-eluting radiopaque embolic beads (BTG-002814) in patients with resectable liver malignancies and to determine concentrations of vandetanib and the N-desmethyl metabolite in plasma and resected liver following treatment with BTG-002814.

**Methods:**

The VEROnA study is a first-in-human, open-label, single-arm, phase 0, window-of-opportunity study of BTG-002814 (containing 100 mg vandetanib) delivered transarterially, 7 to 21 days before surgery in patients with resectable liver malignancies. Eligible patients have a diagnosis of colorectal liver metastases, or HCC (Childs Pugh A), diagnosed histologically or radiologically, and are candidates for liver surgery. All patients are followed up for 28 days following surgery. Secondary objectives of this study are to evaluate the anatomical distribution of BTG-002814 on noncontrast-enhanced imaging, to evaluate histopathological features in the surgical specimen, and to assess changes in blood flow on dynamic contrast-enhanced magnetic resonance imaging following treatment with BTG-002814. Exploratory objectives of this study are to study blood biomarkers with the potential to identify patients likely to respond to treatment and to correlate the distribution of BTG-002814 on imaging with pathology by 3-dimensional modeling.

**Results:**

Enrollment for the study was completed in February 2019. Results of a planned interim analysis were reviewed by a safety committee after the first 3 patients completed follow-up. The recommendation of the committee was to continue the study without any changes to the dose or trial design, as there were no significant unexpected toxicities related to BTG-002814.

**Conclusions:**

The VEROnA study is studying the feasibility of administering BTG-002814 to optimize the use of this novel technology as liver-directed therapy for patients with primary and secondary liver cancer.

**Trial Registration:**

ClinicalTrial.gov NCT03291379; https://clinicaltrials.gov/ct2/show/NCT03291379

**International Registered Report Identifier (IRRID):**

DERR1-10.2196/13696

## Introduction

### Background

Hepatocellular carcinoma (HCC) is the most common primary cancer of the liver and the fourth most common cause of cancer-related death worldwide [1]. Curative treatment options for early-stage HCC include surgical resection, liver transplantation, and ablative therapies. However, less than 30% of HCC patients are eligible for curative-intent therapies because of large tumor burden, vascular invasion, or poor liver function because of underlying liver disease [2]. For patients with intermediate-stage HCC, the current standard treatment is transarterial chemoembolization (TACE) [3,4]. Although TACE provides a survival advantage over best supportive care, it rarely produces a complete response because of persistence of viable tumor cells and is, therefore, not considered a curative therapy [5].

Colorectal cancer (CRC) is the third most commonly occurring cancer in men and the second most commonly occurring cancer in women worldwide [1]. Approximately 25% of patients present with metastatic CRC (mCRC), and approximately 35% to 55% of CRC patients will develop liver metastases at some point during the course of their disease. Surgical resection is the treatment of choice for liver metastases, offering a potential cure if they can be fully resected [6]. However, only 15% to 20% of patients that develop liver metastases will be resectable at presentation because of presence of multifocal tumors or limited hepatic reserve [7,8]. For patients not suitable for resection, TACE has been shown to be an effective treatment option, potentially offering an improvement in quality of life and overall survival. A systemic review of 13 studies, comprising 850 patients with mCRC treated using drug-eluting beads (DEB) loaded with irinotecan, demonstrated an average response rate of 56.2% by response evaluation criteria in solid tumors (RECIST) and 51.2% by modified RECIST/European Association for the Study of the Liver response criteria. The average overall survival was 16 months [9]. The improvement in quality in life is mainly related to the reduced systemic side effects of the chemotherapy drug [10,11]. DEB-TACE is, therefore, a palliative locoregional treatment option that can be considered for mCRC patients with unresectable liver metastases [12].

Compared with conventional TACE, drug-eluting beads transarterial chemoembolization (DEB-TACE) achieves higher intratumoral concentrations and lower systemic concentrations of the cytotoxic agent, thereby reducing the chemotherapy-related toxicity of treatment [13,14]. Although TACE is usually repeated multiple times, both primary and secondary liver cancers can become resistant to treatment, resulting in local progression [15,16]. There is a need to improve clinical outcomes post TACE by improving the delivery of DEB-TACE and by exploring new targeted anticancer drugs that can be delivered directly to the tumor on preloaded beads [17]. A current limitation in improving the accuracy of embolic administration during DEB-TACE and in understanding how well the treatment reaches its target is the inability to visualize the beads on imaging following local delivery within the liver. A radiopaque (RO) bead has recently become commercially available, which can be visualized with computed tomography (CT) and fluoroscopic imaging [18]. This technology has the advantage of providing intra- and postprocedural confirmation on x-ray or CT of bead location during and after the embolization procedure, enabling real-time adjustments to optimize patient treatment [19]. The RO bead builds on existing DC Bead technology, which utilizes polyvinyl alcohol microspheres to embolize blood vessels and deliver drugs at high concentrations to tumors. The lasting radio-opacity of RO beads means that they are visible on x-ray–based follow-up scans, allowing precise evaluation of the completeness of tumor treatment.

Vandetanib is an inhibitor of the tyrosine kinase activity of vascular endothelial growth factor receptor-2 (VEGFR-2), an endothelial cell receptor for vascular endothelial growth factor (VEGF). It also possesses activity against endothelial growth factor receptor (EGFR) and REarranged during Transfection (RET) tyrosine kinases. Pathological angiogenesis is necessary for the progression of solid, malignant tumors, and inhibition of VEGF-dependent signaling has been identified as a key antiangiogenic strategy [20]. EGFR-dependent signaling is an important pathway contributing to the growth and metastasis of tumor cells, and aberrant EGFR tyrosine kinase activity has been reported in a number of human solid tumors. EGFR tyrosine kinase activity plays a key role in tumor growth and progression, including proliferation, dedifferentiation, and inhibition of apoptosis, metastasis, and angiogenesis [21].

### Objectives

The VEROnA study explores the feasibility of administering a vandetanib-eluting radiopaque embolization bead (BTG-022814) [22]. It is the first time that BTG-002814 has been administered to humans. As TACE can be safely used in the preoperative setting to treat cancers before liver surgery, this study is performed in patients with resectable HCC or colorectal liver metastases before resection surgery. This provides a window of opportunity to measure levels of vandetanib in the resected liver sample as well as to assess the safety and tolerability of BTG-002814 in patients with liver cancer. As tumor recurrence rates for HCC and mCRC after resection are up to 70% [23,24], the development of a preoperative therapy, with the potential to improve long-term disease control, is an important research goal.

## Methods

### Inclusion and Exclusion Criteria

The VEROnA study is conducted and documented in accordance with the Declaration of Helsinki. The VEROnA study (NCT03291379) has been approved by the Health Research Authority London-Chelsea Research Ethics Service Committee (17/LO/00/11) and the Medicines and Healthcare Products Regulatory Agency (Clinical Trials Authorization number 2016-004164-19). The inclusion and exclusion criteria for the VEROnA study are summarized in Textboxes 1 and 2, respectively.

### Overview of Study Design

The VEROnA study is a pilot, open-label, single-arm, phase 0, window-of-opportunity study of DEB-TACE treatment with vandetanib-eluting radiopaque beads (BTG-002814) delivered 7 to 21 days before surgery in patients with resectable liver malignancies. A target size of 6 patients with each primary diagnosis is deemed sufficient to assess safety and drug concentrations in plasma and resected specimens following treatment. As this is a single-arm study, there is no randomization. All patients are followed up for 28 to 32 days following surgery. Patients are recruited from 2 centers in the United Kingdom, and liver surgery is performed at 1 tertiary referral center. Patients are identified from regional multidisciplinary team meetings, which cover 10 referring UK centers.

**Inclusion criteria for the VEROnA study.**

1. Male or female adults (aged ≥18 years)
2. Resectable hepatocellular carcinoma (Child Pugh A, International Normalized Ratio ≤1.5) or resectable liver metastases from colorectal cancer and a candidate for liver surgery
3. Low risk for morbidity and mortality from liver surgery
4. World Health Organization performance status 0-2
5. Adequate hematological function with hemoglobin >90 g/L, absolute neutrophil count >1.5 × 10^9^/L, and platelets >75 × 10^9^/L
6. Adequate liver function with serum bilirubin <1.5 × upper limits of normal (ULN), alanine aminotransferase (ALT; aspartate aminotransferase if ALT is not available) ≤5 x ULN, and alkaline phosphatase <5 × ULN
7. Adequate renal function with serum creatinine ≤1.5 x ULN and calculated creatinine clearance (*glomerular filtration rate*) ≥50 mL/min estimated using a validated creatinine clearance calculation (eg, Cockroft-Gault or Wright formula)
8. Willing to provide blood samples, and tissue samples at surgical resection, for research purposes
9. Willing and able to provide written informed consent

**Exclusion criteria for the VEROnA study.**

1. Any systemic chemotherapy within 3 months of the screening visit or any plan to administer systemic chemotherapy before surgery
2. Previous treatment with transarterial embolization (with or without chemotherapy) of the liver, prior radiotherapy or ablation therapy to the liver, or prior yttrium-90 microsphere therapy
3. Any contraindication to vandetanib according to its local label including:
  - Hypersensitivity to the active substance
  - Congenital long corrected QT (QTc) syndrome
  - Patients known to have a QTc interval over 480 milliseconds
  - Concomitant use of medicinal products known to also prolong the QTc interval or induce torsades de pointes
4. Any contraindication to hepatic artery catheterization or hepatic embolization procedures
5. Women of child-bearing potential not using effective contraception or women who are breast feeding
6. Confirmed allergy to iodine-based intravenous contrast media
7. Patients who cannot have computed tomography, magnetic resonance imaging (MRI), or dynamic contrast-enhanced MRI (according to site policy)
8. Active uncontrolled cardiovascular disease
9. Any comorbid disease or condition or event that, in the investigator’s judgment, would place the patient at undue risk and would preclude the safe use of BTG-002814
10. Levels of potassium, calcium, magnesium, or thyroid-stimulating hormone outside the normal ranges and that in the investigator’s judgment is clinically significant, or other laboratory findings that in the view of the investigator makes it undesirable for the patient to participate in the study
11. Participation in another clinical trial with an investigational product within 4 weeks before the screening visit

### Protocol Treatment With BTG-002814

**Figure 1:**
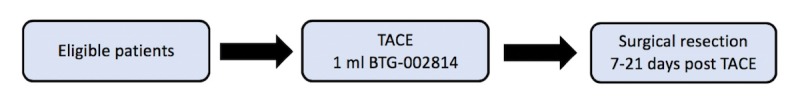
Basic overview of study schema. All eligible patients are treated with 1 mL BTG-002814 (containing 100 mg of vandetanib) via drug-eluting beads-transarterial chemoembolization, 7 to 21 days before surgical resection of the liver tumor or tumors. TACE: transarterial chemoembolization.

Following screening and written consent, all eligible patients receive 1 treatment with BTG-002814, delivered transarterially, 7 to 21 days before surgical resection of the liver (Figure 1). Moreover, 1 vial of BTG-002814, containing 100 mg vandetanib, is used for each patient. To hydrate BTG-002814, 1 mL of water for injection, followed by 9 mL of Omnipaque 350 contrast agent, is added to the vial.

Using a unilateral femoral approach, selective catheterization of the hepatic artery will be performed. Diagnostic visceral arteriography will be performed to delineate the arterial supply to the tumor to determine the presence of variant arterial anatomy and to confirm patency of the portal vein. Once the patient’s arterial anatomy is understood, a catheter will be advanced into the right or left hepatic artery distal to the cystic artery (if visualized). The treatment plan will be based on the fluoroscopic appearances during arteriography. Once the catheter is in place within the artery feeding the tumor, the reconstituted BTG-002814 suspension will be slowly infused into the artery (approximately 1 mL per minute). The end point of the procedure is either full delivery of the reconstituted bead volume (ie, 1 mL vandetanib loaded beads in contrast) or near-stasis in the tumoral vessel over 6 cardiac cycles. Undelivered volume of the reconstituted embolic solution will be recorded. For HCC patients, a super selective (segmental/subsegmental) approach will be taken with the catheter placed as selectively as possible while maintaining sufficient flow to the tumor. For mCRC patients, a lobar approach will be taken.

### Study Objectives

The primary aim of the VEROnA clinical study is to assess the safety and tolerability of vandetanib-eluting radiopaque embolic beads (BTG-002814).

#### Coprimary End Points

Coprimary end points of this study are as follows:

- Adverse events (AEs) related to treatment with BTG-002814 using the standardized grading criteria (National Cancer Institute Common Terminology Criteria for Adverse Events Version 4.0 [NCI-CTCAE v4.0]).
- Concentration of vandetanib and N-desmethyl vandetanib in plasma and in resected liver tissue following treatment with BTG-002814.

#### Secondary End Points

Secondary end points of this study are as follows:

- Distribution of BTG-002814 on noncontrast-enhanced imaging of tumor vasculature and regions of interest using 4-dimensional (4D) CT.
- Evaluation of histopathological features in the surgical specimen (malignant and nonmalignant liver tissue): tumor necrosis, viable tumor, and vascular changes.
- Assessment of changes in blood flow on dynamic contrast-enhanced magnetic resonance imaging (DCE-MRI) following treatment with BTG-002814.

#### Exploratory End Points

Exploratory end points of this study are as follows:

- Study blood biomarkers with the potential to identify patients likely to respond to treatment with BTG-002814
- Correlate the distribution of BTG-002814 on imaging and pathology by 3-dimensional (3D) modeling.

### Study Schedule

**Figure 2:**
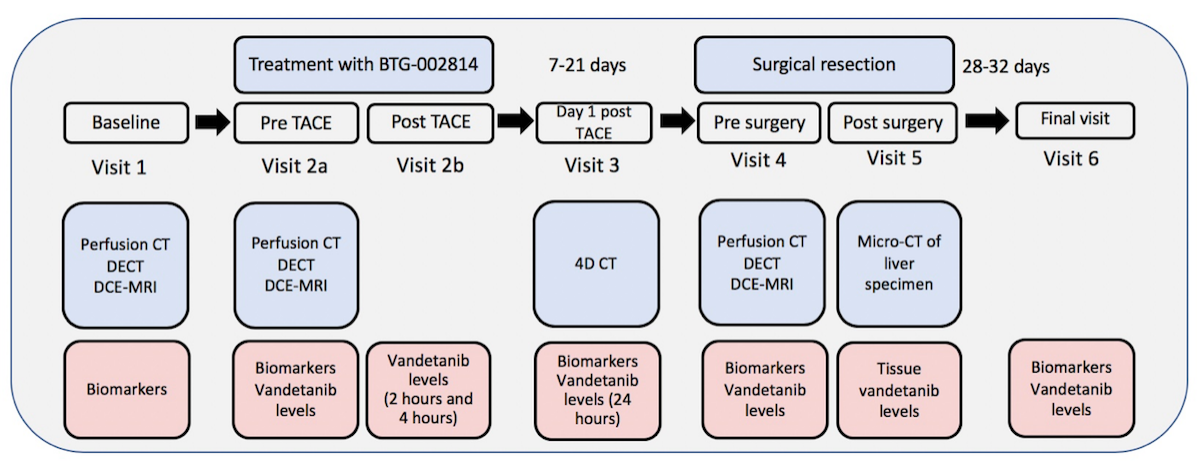
Study schema for the VEROnA study. CT: computed tomography; DCE-MRI: dynamic contrast-enhanced–magnetic resonance imaging; DECT: dual energy computed tomography; 3D: 3-dimensional; 4D: 4-dimensional; TACE: transarterial chemoembolization.

Figure 2 shows an overview of the study schema. A baseline visit is performed up to 7 days before treatment with BTG-002814. This visit involves liver magnetic resonance imaging (MRI) scan (incorporating dynamic contrast-enhanced–MRI [DCE-MRI]) and liver CT scan (incorporating perfusion CT liver, pCT), in addition to blood samples for biomarkers. On the day of treatment with BTG-002814, pretreatment assessments include liver DCE-MRI and pCT, blood biomarker analysis, and plasma vandetanib and metabolite analysis. Following treatment, blood samples for vandetanib and metabolite analysis at 2 and 4 hours are taken. Patients are admitted overnight for observation. A noncontrast 4-dimensional CT scan is performed the following day to assess the location of beads. Blood samples are taken for biomarker and vandetanib and metabolite analysis 24 hours post treatment. Imaging with DCE-MRI and pCT is repeated on the day before surgical resection, along with blood biomarker, vandetanib, and metabolite analysis. Surgical resection of the liver tumor(s) is then conducted as part of standard clinical care. Following resection, the specimen is imaged in a micro-CT scanner before sampling for tissue vandetanib levels. Histopathological analysis is then performed. Table 1 outlines the assessments undertaken at each study visit.

**Table 1:** Outline of study schedule and assessments.

Study visit and assessment	Visit 0: screening	Visit 1: baseline	Visit 2: treatment day		Visit 3: day 1 posttreatment	Visit 4: day before resection	Visit 5: surgical resection	Visit 6: end of study
			Pre	Post				
Informed consent	X^a^	—^b^	—	—	—	—	—	—
Patient demographics	X	—	—	—	—	—	—	—
Medical and prior treatment history	X	—	—	—	—	—	—	—
Eligibility assessment	X	—	—	—	—	—	—	—
Child Pugh Assessment (hepatocellular carcinoma patients only)	X	—	—	—	—	—	—	—
Physical examination	X	X	—	—	—	X	—	X
Vital signs	X	X	X	X	X	X	X	X
World Health Organization performance status	X	X	—	—	—	X	—	X
Concomitant medications	X	X	X	X	X	X	X	X
Assessment of adverse events	X	X	X	X	X	X	X	X
Biochemistry	X	X	—	—	X	X	—	X
Hematology	X	X	—	—	X	X	—	X
Coagulation tests	X	X	—	—	—	X	—	X
12-lead electrocardiogram	X	X	X	X	—	X	—	X
Serum pregnancy test^c^	X	X	—	—	—	—	—	—
Liver MRI^d^ (incorporating dynamic contrast-enhanced–MRI)	—	X	X	—	—	X	—	—
CT^e^ scan liver (incorporating perfusion sequence)	—	X	X	—	—	X	—	—
4-dimensional CT scan liver	—	—	—	—	X	—	—	—
Blood biomarker analysis	—	X	X	—	X	X	—	X
Serum tumor markers	—	X	X	—	—	X	—	—
Vandetanib and N-desmethyl metabolite plasma sampling	—	—	X	X	X	X	—	X
Vandetanib and N-desmethyl metabolite tissue sampling and histopathological diagnosis	—	—	—	—	—	—	X	—

^a^Assessment performed at visit.

^b^Assessment not performed at visit.

^c^For women of child-bearing potential, a negative pregnancy test must be obtained before treatment.

^d^MRI: magnetic resonance imaging.

^e^CT: computed tomography.

### Safety and Tolerability of BTG-002814

AEs and serious AEs are collected and classified according to standardized grading criteria (NCI-CTCAE v4.0) and the relationship to the study treatment recorded.

### Plasma and Tissue Levels of Vandetanib and N-Desmethyl Metabolite

Vandetanib is metabolized to its major metabolite, N-desmethyl vandetanib, by cytochrome P450 3A4 (CYP3A4). Concentrations of vandetanib and N-desmethyl vandetanib in plasma and in resected liver tissue following treatment with BTG-002814 are measured and concentration profiles over time are reported.

Following surgical resection, the liver specimen is immediately secured within a plastic bag and stored on ice in an insulated container before transfer for sampling. Tissue samples are collected from the resected liver tissue to measure concentrations of vandetanib and N-desmethyl vandetanib. Samples are immediately wrapped in foil, snap frozen into liquid nitrogen, and stored at −80°C until shipped on dry ice to York Bioanalytical Solutions Ltd, York, United Kingdom, for analysis.

Blood samples for vandetanib concentrations are taken before treatment with BTG-002814 (0 min) and at 2 hours, 4 hours, and 24 hours following initial administration. If patients require a longer hospital stay, an optional additional sample is taken after 36 hours and up to the time of hospital discharge. Samples are then taken 24 hours before surgery (visit 4) and at the end of study visit (visit 6; Figure 2). At each time point for analyses, whole blood is collected into a lithium heparin tube and centrifuged. Plasma is pipetted into cryovials, which are stored at −70°C (or below) until analysis (York Bioanalytical Solutions Ltd, United Kingdom). Vandetanib and N-desmethyl vandetanib metabolite concentrations in plasma and tissue samples are determined using solid phase extraction, followed by liquid chromatography coupled to mass spectrometry (York Bioanalytical Solutions Ltd, York, United Kingdom).

### Distribution of BTG-002814

Following treatment with BTG-002814, a 4D-CT scan is performed to assess the positioning of the beads. This scan is performed without contrast and is used to describe the anatomical distribution of the RO beads.

### Histopathology of Resected Liver

Following liver resection, an evaluation of histopathological features in both malignant and nonmalignant liver tissue from the surgical specimen is performed. The extent of tumor necrosis, viable tumor, and any vascular changes observed are described. Conventional techniques will be used to further investigate the tumor environment, such as extent of necrosis and neoangiogenesis.

### Dynamic Contrast-Enhanced–Magnetic Resonance Imaging and Perfusion Computed Tomography Imaging

As measurement of perfusion characteristics may improve understanding of liver tumor biology and behavior, patients in this study undergo DCE-MRI and perfusion CT (pCT) at time points shown in Figure 2.

#### Dynamic Contrast-Enhanced–Magnetic Resonance Imaging

After acquisition of standard clinical MRI liver sequences, T1 mapping is performed using 3D volumetric gradient echo imaging with varying flip angles. A series of T1-weighted 3D volumetric images are acquired at baseline and at short intervals during administration of a bolus of intravenous paramagnetic MR contrast agent. Liver parenchyma and tumor signal intensity curves are used to calculate tissue parameters describing tumor perfusion, blood flow, and vascularity using a compartment model with an arterial and portal venous input function [25].

#### Dual Energy and Perfusion Computed Tomography

Dual energy CT (DECT) imaging and perfusion imaging (pCT) of the liver are performed. A DECT (80 and 135 kV) and helical CT (120 kV) is acquired before contrast delivery. Following injection of iodinated contrast, volume perfusion scans are acquired. Dual energy and helical CT scans are then repeated following contrast washout. CT perfusion maps are created and used to calculate tissue parameters describing tumor perfusion, blood flow, and vascularity. Arterial blood flow, portal venous blood flow, and perfusion index will be derived using a dual input maximum slope method; blood volume and flow extraction product will be derived using Patlak analysis [26].

### Blood Biomarkers

Blood biomarkers are measured to indicate potential activity of the BTG-002814. Specifically, proangiogenic factors, antiangiogenic factors, hypoxia markers, hemopoetic growth factors, inflammatory factors, and markers of endothelial function are measured at set time points during the study (Figure 2). At each study time point, blood is collected into an EDTA tube and centrifuged. Plasma is pipetted as aliquots into 6 to 8 cryovials, which are stored at −70°C (or below). Measurements of each cytokine is then performed using the Merck Milliplex kits for the Luminex 200 machine. Blood samples are also taken for the measurement of serum alpha-fetoprotein in patients with HCC, or serum CEA, CA19-9, and CA125 in patients with mCRC.

### Three-Dimensional Modeling

The correlation between the distribution of BTG-002814 on imaging and within the pathological specimen will be explored using 3D modeling. Following resection of the tumor, micro-CT imaging is performed on a Mediso nanoScan Positron Emission Tomography/CT. After micro-CT, standard hematoxylin and eosin stain sections are cut from every block onto large microscopy slides. All slides are scanned and image sets are uploaded and registered using a sequential slice-to-slice image-based registration approach.

### Statistical Considerations

The statistical analysis in this study is primarily descriptive, to assess the safety and tolerability of the study treatment as well as the distribution of the product following delivery. As such, the study is not powered for any statistical hypotheses. On the basis of preclinical trials, a target size of 6 patients with each primary diagnosis is deemed sufficient to assess safety and drug concentrations in plasma and resected specimens following treatment [27]. All patients who receive treatment with BTG-002814 in the study will be included in the analysis population.

## Results

Enrollment for the study will be completed in February 2019. Results of a planned interim analysis were reviewed by a safety committee after the first 3 patients completed follow-up. The recommendation of the committee was to continue the study without any changes to the dose or trial design, as there were no significant unexpected toxicities related to BTG-002814.

## Discussion

### Overview

Although surgical treatment is the treatment of choice for HCC and CRC liver metastases, only a proportion of patients are suitable for surgery because of the presence of multifocal tumors or limited hepatic reserve [6]. TACE is the standard treatment option for intermediate-stage HCC and is now considered a local therapy option for some patients with liver-limited mCRC. Despite recent advances in TACE delivery, including the development of DEB-TACE, tumor progression occurs in the majority of patients [15,16]. Improving administration techniques and investigating the local delivery of new anticancer drugs via TACE are important research approaches to potentially improving clinical outcomes for patients with primary and secondary liver cancer.

The ability to deliver vandetanib locally to liver tumors via TACE is a novel approach to be reported in this first-in-human clinical trial. To assess targeting of angiogenesis (via VEGF) and EGFR- and RET-dependent tumor cell growth, anticancer efficacy will be assessed in this clinical trial primarily by the extent of tumor necrosis and percentage of viable tumor in the resected liver tissue, in addition to the measurement of relevant blood biomarkers.

To date, there has been only 1 trial that has assessed the efficacy of vandetanib in HCC patients. That study, by Hsu et al, involved oral systemic administration rather than transarterial targeted therapy. A total of 67 HCC patients were randomized to oral vandetanib 300 mg (n=19), oral vandetanib 100 mg (n=25), or placebo (n=23). A total of 29 patients subsequently entered open-label treatment. Vandetanib induced a significant increase in circulating VEGF and a decrease in circulating VEGFR levels. In both vandetanib treatment arms, tumor stabilization rate was not significantly different from placebo. Although trends toward improved progression-free survival and overall survival after vandetanib treatment were found, they were not statistically significant. The most common AEs were diarrhea and rash in both treatment groups [28].

In patients with mCRC, several phase I dose escalation studies have been conducted to determine the maximum tolerated dose of oral vandetanib in combination with different therapeutic agents and regimens. These studies have reported expected and manageable toxicity profiles, but the observed efficacies have raised concern for moving forward with these combinations [29-33].

It is anticipated that the tissue concentrations of vandetanib and its major metabolite will be significantly higher in liver tumors following administration of TACE beads than following oral administration. One would speculate that the systemic concentrations of the drug should be lower following local delivery to the liver compared with oral administration, resulting in a more tolerable side effect profile, as observed in a pharmacokinetic study of BTG-002814 in a porcine model of hepatic artery embolization [27]. In this preclinical study, healthy swine were treated with intra-arterial vandetanib-eluting radiopaque beads, and tissue samples were taken from both embolized and nonembolized liver sections for determination of vandetanib and metabolite levels at necropsy (30 and 90 days). Vandetanib and N-desmethyl vandetanib were present in treated sections 30 days after administration, at levels above the in vitro IC_50_ for biological effectiveness. At 90 days, both analytes were still present in the treated liver sections but were near or below the limit of quantification in untreated liver sections, demonstrating sustained release from the loaded beads. Furthermore, intra-arterial delivery resulted in low systemic exposure, with no obvious systemic toxicity. On the basis of these data, it is postulated that, in this first-in-human study, BTG-002814 will be safe to deliver, provide sustained release of vandetanib, and have minimal toxicity from systemic exposure to drug.

### Conclusions

The VEROnA study is a first-in-human, window-of-opportunity study of the safety and tolerability of BTG-002814 in patients with resectable HCC or mCRC. The study design allows translational end points to be evaluated in detail, including blood biomarkers, perfusion imaging studies, and 3D histopathological modeling.

## Abbreviations

3D: 3-dimensional
4D: 4-dimensional
AE: adverse event
CRC: colorectal cancer
CT: computed tomography
DEB: drug-eluting beads
DECT: dual energy computed tomography
EGFR: endothelial growth factor receptor
HCC: hepatocellular carcinoma
mCRC: metastatic colorectal cancer
NCI-CTCAE v4.0: National Cancer Institute Common Terminology Criteria for Adverse Events Version 4.0
pCT: perfusion computed tomography
RECIST: response evaluation criteria in solid tumors
RET: REarranged during Transfection
RO: radiopaque
TACE: transarterial chemoembolization
VEGF: vascular endothelial growth factor
VEGFR: vascular endothelial growth factor receptor
